# Retrospective Analysis of Bacterial Cultures Sampled in German Chicken-Fattening Farms During the Years 2011–2012 Revealed Additional VIM-1 Carbapenemase-Producing *Escherichia coli* and a Serologically Rough *Salmonella enterica* Serovar Infantis

**DOI:** 10.3389/fmicb.2018.00538

**Published:** 2018-03-27

**Authors:** Nicole Roschanski, Jennie Fischer, Linda Falgenhauer, Michael Pietsch, Sebastian Guenther, Lothar Kreienbrock, Trinad Chakraborty, Yvonne Pfeifer, Beatriz Guerra, Uwe H. Roesler

**Affiliations:** ^1^Institute for Animal Hygiene and Environmental Health, Freie Universitaet Berlin, Berlin, Germany; ^2^Department for Biological Safety, Federal Institute for Risk Assessment, Berlin, Germany; ^3^German Center for Infection Research, Institute of Medical Microbiology, Justus Liebig University Giessen, Partner Site Giessen-Marburg-Langen, Giessen, Germany; ^4^FG13 Nosocomial Pathogens and Antibiotic Resistance, Robert Koch Institute, Wernigerode, Germany; ^5^Epidemiology and Information Processing and WHO Collaborating Center for Research and Training for Health at the Human-Animal-Environment Interface, Institute for Biometry, University of Veterinary Medicine Hannover, Hannover, Germany

**Keywords:** antimicrobial resistance, plasmids, livestock, carbapenems, VIM-1 producing Enterobacteriaceae, Germany, broiler

## Abstract

Carbapenems are last-resort antibiotics used in human medicine. The increased detection of carbapenem-resistant Enterobacteriaceae (CRE) is therefore worrying. In 2011 we reported the first livestock-associated VIM-1-producing *Salmonella* (*S*.) *enterica* serovar Infantis (R3) isolate from dust, sampled in a German chicken fattening farm. Due to this observation we retrospectively investigated more than 536 stored bacterial cultures, isolated from 45 chicken fattening farms during the years 2011 and 2012. After a non-selective overnight incubation, the bacteria were transferred to selective media. *Escherichia (E.) coli* and *Salmonella* growing on these media were further investigated, including antibiotic susceptibility testing, carbapenemase gene screening and whole genome sequencing (WGS). In total, four CRE were found in three out of 45 investigated farms: Besides R3, one additional *Salmonella* (G-336-1a) as well as two *E. coli* isolates (G-336-2, G-268-2). All but G-268-2 harbored the *bla*_VIM-1_ gene. *Salmonella* isolates R3 and G-336-1 were closely related although derived from two different farms. All three *bla*_VIM-1_-encoding isolates possessed identical plasmids and the *bla*_VIM-1-_ containing transposon showed mobility at least *in vitro*. In isolate G-268-2, the AmpC beta-lactamase gene *bla*_CMY-2_ but no known carbapenemase gene was identified. However, a transfer of the phenotypic resistance was possible. Furthermore, G-268-2 contained the *mcr-1* gene, combining phenotypical carbapenem- as well as colistin resistance in one isolate. Carbapenem-resistant Enterobacteriaceae have been found in three out of 45 investigated chicken flocks. This finding is alarming and emphasizes the importance of intervention strategies to contain the environmental spread of resistant bacteria in animals and humans.

## Introduction

Carbapenem resistance increased in many countries during the last years, causing serious problems in the public health system (Wilson, 2017). As carbapenems serve as one of the last remaining options for the treatment of serious infections caused by multidrug-resistant Gram-negative bacteria (e.g., Enterobacteriaceae, *Acinetobacter baumannii, Pseudomonas aeruginosa*), the recent situation is alarming. In terms of Gram-negative bacteria, mainly the expression of carbapenemases leads to a decreased carbapenem-susceptibility. Most frequently detected genes are *bla*_VIM_, *bla*_IMP_, *bla*_NDM_ (class B ß-lactamases), *bla*_KPC_ (class A β-lactamases) and *bla*_OXA−48−like_ (class D β-lactamases) (Nordmann et al., 2012). Their location on mobile genetic elements though contributes to a successful spread of these resistance genes (Nordmann et al., 2012; Carattoli, 2013). Within the last couple of years, the occurrence of carbapenemase-producing bacteria relocated from clinical settings. Carbapenemase-producing bacteria have been isolated from the environment (Walsh et al., 2011; Zurfluh et al., 2013), wild-birds (Fischer et al., 2013b), seafood (Rubin et al., 2014; Morrison and Rubin, 2015; Roschanski et al., 2017b), companion- and food-producing animals all over the world (Stolle et al., 2013; Mollenkopf et al., 2016; Ewers et al., 2017; Fischer et al., 2017; He et al., 2017). In terms of food-producing animals, beside pigs and cattle another focus was chicken and chicken meat. In 2011, the first VIM-1 producing *Salmonella enterica* subsp. *enterica* serovar Infantis (*S*. Infantis)—isolate R3—was isolated on a German chicken fattening farm (Fischer et al., 2013a). This isolate was closely related to other *S*. Infantis isolates detected in tree pig fattening farms in the same year (Fischer et al., 2017), based on identical macrorestriction patterns and the presence of a *bla*_VIM-1_-carrying IncHI2 plasmid of 300 kb. Therein, the carbapenemase gene was located within a class 1 integron embedded in a Tn*21* homolog (Falgenhauer et al., 2017). Since 2015, additional publications described the finding of carbapenemase-producing bacteria in chicken or retail chicken meat. Carbapenemase-producing Enterobacteriaceae (CPE) were detected in retail chicken meat and in broiler farms in Egypt (Abdallah et al., 2015), and quite recently, the prevalence of NDM and Mcr-1 in Chinese poultry production as well as VIM-positive *Pseudomonas* species in Chinese chicken and their surrounding has been described (Wang et al., 2017; Zhang et al., 2017). To follow-up on the study of Fischer et al. (Fischer et al., 2013a), 536 stored bacterial cultures, isolated from 45 chicken-fattening farms as well as 125 stored single colony cultures derived from the previously *bla*_VIM-1_-positive chicken farm 1, were retrospectively investigated for the presence of carbapenem-resistant *E. coli* as well as *Salmonella* isolates.

## Materials and methods

### Bacterial cultures and screening for carbapenem-resistant isolates

#### Primary mixed bacterial cultures

In total, 536 primary bacterial cultures, isolated from pooled feces, pooled dust as well as boot swab samples were retrospectively investigated. The samples were initially taken in terms of the first period of the national research project RESET (www.reset-verbund.de), carried out during the years 2011–2013, and focused on screening for ESBL and AmpC-producing Enterobacteriaceae in different habitats (Laube et al., 2013; Hering et al., 2016). Therein, an overall number of 45 chicken fattening farms distributed throughout Germany have been investigated. Briefly, the samples were taken from each farm, incubated non-selectively in LB broth (Luria/ Miller), (Carl Roth, Karlsruhe, Germany), followed by a selective cultivation on MacConkey agar plates containing 1 mg/L cefotaxime (293 cultures – 55%) or Endo-agar containing 2 mg/L enrofloxacin (243 cultures – 45%). Mixed sets of bacteria, able to grow on these plates (primary cultures), were taken from the plates and stored in Cryobank™ (MAST Diagnostica, Reinfeld, Germany) at −80°C (Laube et al., 2013; Hering et al., 2016). For our retrospective analyses, the stored bacteria were re-cultured non-selectively in LB broth at 37°C, overnight. Each culture was spread on selective MacConkey agar plates (MacConkey agar No 3; OXOID, Hampshire, UK) containing 0.125 mg/L meropenem, (Sigma Aldrich, Seelze, Germany). *E. coli* and *Salmonella* colonies were isolated and species were confirmed using MALDI-TOF mass-spectrometry (MALDI Microflex®LT and Biotyper®database, Bruker Daltronics, Bremen, Germany). Per sample and species, one colony was picked and subsequently spread on chromID™-CARBA agar plates (bioMérieux, Nurtingen, Germany). Thereon grown colonies (one per sample and species) were further investigated for the presence of carbapenemase genes (*bla*_VIM_, *bla*_KPC_, *bla*_NDM_, *bla*_OXA-48_, and *bla*_GES_) using real-time PCR (Roschanski et al., 2017a). The presence of *bla*_IMP_ was checked in a conventional PCR format (van der Zee et al., 2014).

#### Single colonies derived from chicken-farm 1

In a separate investigation, performed in the department of biological safety of the Federal Institute for Risk Assessment, 120 stored single colony cultures (111 *E. coli*, 5 *Pseudomonas*, 4 *Acinetobacter*), derived from the previously VIM-positive tested chicken farm 1 (*S*. Infantis R3), were screened for the presence of further *bla*_VIM-1_-encoding isolates. The cultures derived from one of the seven investigated chicken fattening farms which were sampled in terms of a longitudinal study (three samplings per fattening period) conducted by the FU Berlin (Laube et al., 2013).

The cultures were taken from the −80°C stock and non-selectively re-cultured. On the following day the overnight cultures were transferred to LB broth containing 1 mg/L imipenem (1:500). In case of bacterial growth, cultures were diluted 1:10 and used as template for amplification of the *bla*_VIM-1_ gene by PCR. Cultures tested positive by PCR were subsequently spread on selective agar plates for single colony isolation and their *bla*_VIM-1_ confirmation by PCR as previously described (Fischer et al., 2017).

### Antimicrobial susceptibility testing and blue-carba assay

Minimal inhibitory concentrations for the wildtypes as well as the transformants were determined by using the VITEK-2® compact system and the AST-card N248 (bioMérieux, Nuertingen, Germany). The Blue-Carba assay for confirmation of carbapenemase activity was performed in two technical replicates as previously described (Pires et al., 2013).

### Genotypic investigation of wildtype and transformant isolates

Plasmids were isolated using the NucleoBond Xtra Midi kit (Macherey-Nagel, Dueren, Germany) and transferred into *E. coli* NEB®10-beta or NEB®5-alpha (NEB, Frankfurt a. M., Germany) by electroporation (2.5 kV). WGS was performed for the wildtype strains (*Salmonella* R3 and G-336-1a; *E. coli* G-336-2 and G-268-2) as well as the G-336-1a- and G-336-2-transformants T_G-336-1*a*_, T_G-336-2_VIM_, T_G-336-2_CMY+VIM_, T_G-336-2_CMY_ using MiSeq (Illumina) (Borowiak et al., 2017). In addition, the *E. coli* recipient strain NEB®10-beta was sequenced. The raw-data were *de novo* assembled using SPAdes (Bankevich et al., 2012). An additional assembly was performed for G-268-2 by A5-miseq (v. 0.0.9 beta; default parameters) using trimmed raw reads (Trimmomatic: v. 0.0.9; default parameters except maxinfo 15:0.5) (Bolger et al., 2014; Coil et al., 2015). Resistance genes, virulence genes, plasmid incompatibility groups as well as multilocus sequence types were identified using the Web-tools ResFinder (Zankari et al., 2012), PlasmidFinder (Carattoli et al., 2014) and MLST 1.8 (Larsen et al., 2012). In addition, the Resistance Gene Identifier (RGI) was used (Jia et al., 2017). The genetic relationship of the two *S*. Infantis isolates R3 and G-336-1a was determined by using the CSI Phylogeny-1.4 Server (Kaas et al., 2014). Therefore the raw reads were uploaded and mapped against a *S*. Infantis reference sequence (LN649235.1), (Olasz et al., 2015). Parameters were set as follows: Minimum depth at SNP position: 10, relative depth at SNP position: 10, minimum distance between SNPs (prune): 10, minimum SNP quality: 30, minimum read mapping quality: 25 as well as minimum Z-score: 1.96.

A prediction of Inc RNA folding was performed using the RNAfold WebServer (http://rna.tbi.univie.ac.at//cgi-bin/RNAWebSuite/RNAfold.cgi) (Gruber et al., 2008). Furthermore, a subsequent plasmid comparison was performed using the BLAST Ring Image Generator (BRIG) (Alikhan et al., 2011). For this purpose contigs containing plasmid sequences were plotted against two already published *bla*_VIM-1_ containing IncHI2-plasmid sequences derived from a *S*. Infantis as well as an *E. coli* isolated on a German pig fattening farm (pRH-R27, LN555650.1 and pRH-R178 HG530658.1) (Falgenhauer et al., 2017). Whole genome data of all isolates have been deposited in the European Nucleotide Archive (https://www.ebi.ac.uk/ena) of the European Bioinformatics Institute (EMBL-EBI); accession numbers R3 (ERS2154041), G-336-1a (ERS2101552), G-336-2 (ERS2101553), G-268-2 (1969-10-8; ERS2101554), NEB®10-beta (ERS2101551), T_G-336-1*a*_ (FU11995; ERS2101550), T_G-336-2_VIM_ (FU12739; ERS2101547),T_G-336-2_CMY+VIM_ (FU12738; ERS2101548), T_G-336-2_CMY_ (FU11994; ERS2101549).

The phenotypic carbapenem resistance of G-268-2 as well as its transformant T_G-268-2_ was further characterized by an additional screening for the presence of the outer-membrane protein genes *omp*C and *omp*F. Therefore, the whole genome reads were mapped against respective *E. coli* K-12 MG1655 (NC_000913.3) reference sequences. In addition, a PCR-based screening was performed, using the primer pairs Ec_OmpC-fwd – ATGAAAGTTAAAGTACTGTCCCTCC, Ec_OmpC-rev. – TTAGAACTGGTAAACCAGACCCA (1,150 bp), Ec_OmpF-fwd. – ATGATGAAGCGCAATATTCTGG, Ec_OmpF-rev. – TTAGAACTGGTAAACGATACCCACA (1,190 bp) as well as primers described by Lartigue et al. (2007).

### Classical bacterial strain typing

Classification of the *E. coli* isolates into one of the eight described phylogenetic groups was done in accordance to the protocol of Clermont et al. (2013). Serotyping of *Salmonella* isolates was performed in the German National Reference Laboratory for the Analysis and Testing of Zoonoses (NRL Salmonella - BfR, Berlin) according to the White-Kauffmann-Le Minor scheme (Grimont and Weill, 2007). Genetic relatedness of the *Salmonella* isolates was analyzed using XbaI-restriction of bacterial DNA and subsequent pulsed-field gel electrophoresis (PFGE) (Ribot et al., 2006). PFGE was conducted using the CHEF-DR III system (Bio-Rad Laboratories GmbH, Munich, Germany) using a 1.1% agarose gel (Biozyme LE GP agarose; Biozym Scientific GmbH, Hessisch Oldendorf, Germany). The following conditions were used: Initial switch time 5 s, final switch time 50 s at a gradient of 5.6 V/cm and an included angle of 120 V. The run time was 21 h at a system temperature of 14°C. For plasmid characterization, S1-nuclease restriction and PFGE (Guerra et al., 2004) was performed using the following running conditions: 1-25 s, 17 h, 6 V/cm, 120 V.

## Results

### Occurrence of carbapenem-resistant isolates within german chicken fattening farms

In 2011 the first VIM-1-producing *S*. Infantis (R3) was isolated from dust sampled on a German chicken fattening farm. The primary mixed bacterial culture from this chicken farm was included in this study and served as an internal identification control for the applied isolation procedure of CPE. Besides R3, one additional *Salmonella* isolate, serologically typed as subspecies I (rough phenotype, G-336-1a) as well as two *E. coli* isolates (G-336-2, G-268-2) were isolated from the selective agar plates. The real-time PCR-based screening of the new isolates indicated the presence of the *bla*_VIM-1_ gene in G-336-1a and G-336-2. Both of them were found in one dust sample from a chicken fattening farm in South Germany. Apparently, there was no regional connection to the previously described chicken fattening farm (isolate R3) which was located in the eastern part of Germany. G-268-2 was isolated from a boot swab sample originated from a third chicken fattening farm located in East Germany. For this *E. coli* isolate none of the six investigated carbapenemase genes (*bla*_VIM_, *bla*_KPC_, *bla*_NDM_, *bla*_OXA−48_, *bla*_GES_, and *bla*_IMP_) was detected.

### Detailed characterization of *bla*_VIM-1_ containing *Salmonella* and *E. coli* isolates

**Figure 1:**
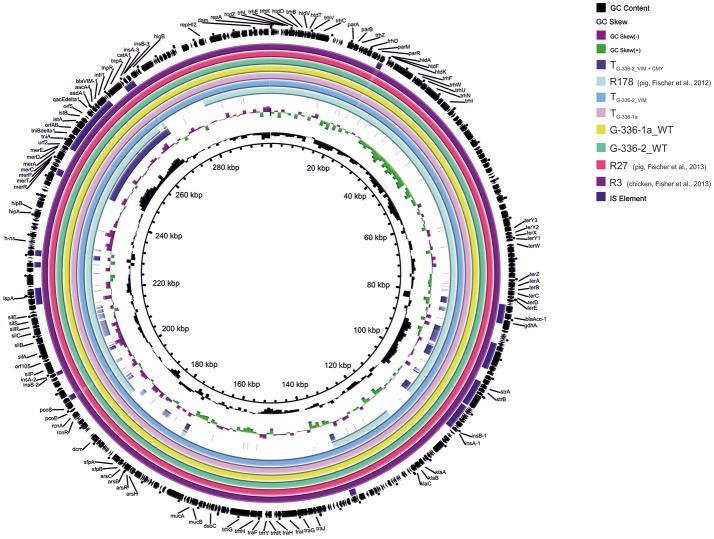
Circular visualization and comparison of *bla*_VIM-1_-carrying IncHI2 plasmid sequences from *S*. Infantis as well as *E. coli* isolates derived from German chicken- as well as pig fattening farms and their transformants using BRIG. WT, wildtype; T, transformed *E. coli* NEB10®-beta.

**Figure 2:**
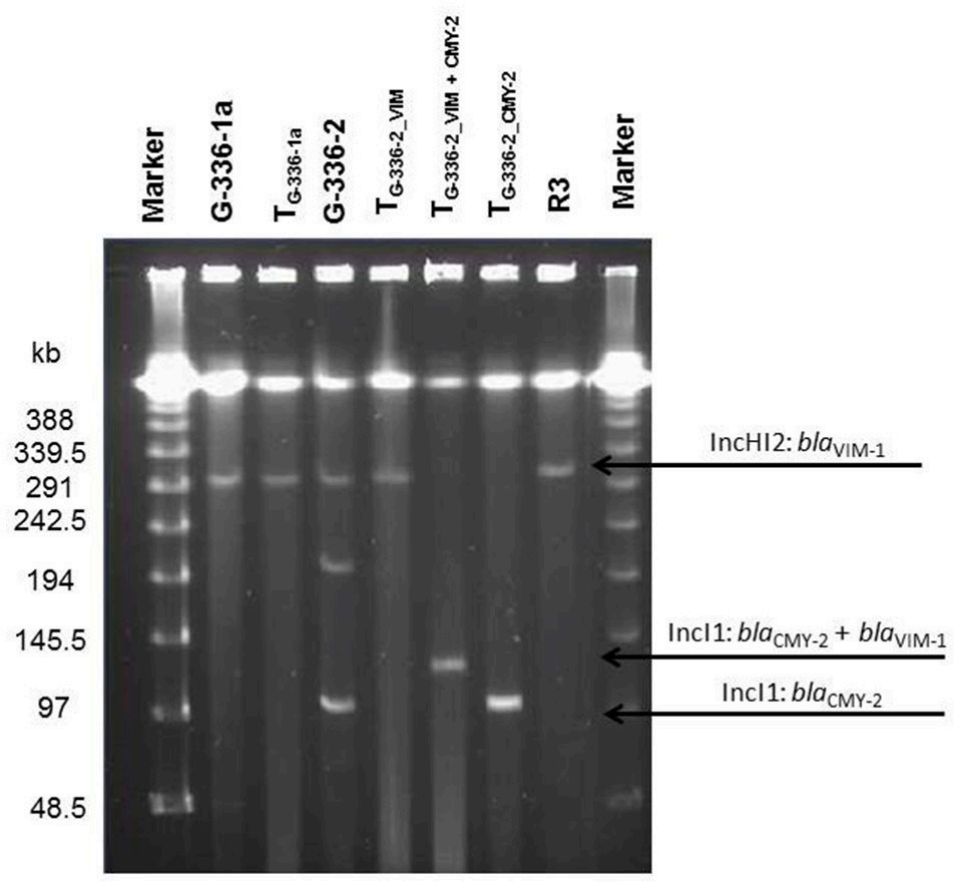
Plasmid content of donor strains and transformants (S1-PFGE). Information about respective replicon types and the resistance gene localization derived from the whole genome data analysis. WT, wildtype; T, transformed *E. coli* NEB10®-beta.

An overview of the whole genome results is provided in Table 1. These data confirm the relatedness of both *Salmonella* isolates (R3 and G-336-1). They belonged to the multilocus sequence type ST32 and both, PFGE as well as SNP analysis, showed only small differences of 2 bands (data not shown) and 8 SNPs, respectively. These data reveal that G-336-1 just as R3, genotypically belongs to the serovar Infantis. The *bla*_VIM-1_-encoding plasmids (IncHI2; 300 kb) derived from the two *S*. Infantis isolates (R3, G-336-1a) as well as the *E. coli* (G-336-2) were compared with the IncHI2 plasmid sequences of *E. coli* R178 as well as *S*. Infantis R27, both isolated in a German pig-fattening farm (Falgenhauer et al., 2017). An identity of 100% was detected between the plasmids of R3, G-336-1a and R27 (*Salmonella*) as well as the *bla*_VIM-1_ containing plasmid of *E. coli* G-336-2 (Figure 1). In all four plasmids the *bla*_VIM-1_ gene was part of a class 1 integron accompanied by the genes *aac*A4 and *aad*A1 in its variable region. As described previously, the integron was inserted in a Tn*21* homolog (Falgenhauer et al., 2017). On the same plasmid, the AmpC gene *bla*_ACC-1_ was detected. S1-PFGE indicated that *E. coli* isolate G-336-2 (sequence type ST131) contained two additional plasmids, one of them an IncI1 plasmid, carrying the AmpC gene *bla*_CMY-2_ (Figure 2). Proofed by WGS, it was possible to show that in course of *in vitro* cultivation and transformation experiments, one *E. coli* transformant was received, which contained the *bla*_VIM-1_-encoding transposon integrated into the *pilU* gene of the *bla*_CMY-2_-encoding IncI1 plasmid (Figure 2). This indicates that at least *in vitro* the transposon is highly mobile and self-transmissive.

**Table 1:** Characteristics of carbapenem-resistant *E. coli* and *S*. Infantis isolates including their respective transformants derived from whole genome data analyses.

**Wildtype-isolate/transformants**	**Farm no**.	**Species [MLST]**	**Resistance genes**	**Plasmids [pMLST]**
R3^*^	1	*Salmonella* Infantis [ST-32]	*aac(6′)Ib*-cr-like, *aac*A4-like, *aad*A1, *bla*_ACC-1_, ***bla***_**VIM−1**_, *cat*A1-like, *ere*(A)-like, *str*A, *str*B, *sul*1-like	IncHI2 [ST-1]
G-336-1a	78	*Salmonella* subspecies I [ST-32]	*aac(6′)Ib*-cr-like, *aac*A4-like, *aad*A1, *bla*_ACC-1_, ***bla***_**VIM−1**_, *cat*A1-like, *str*A, *str*B, *sul*1-like	IncHI2 [ST-1]
T_G-336-1*a*_	–	*E. coli* [ST-1060]	*aac(6′)Ib*-cr-like, *aac*A4-like, *aad*A1, *bla*_ACC-1_, ***bla***_**VIM−1**_, *cat*A1-like, *str*A, *str*B, *sul*1-like	IncHI2 [ST-1]
G-336-2	78	*E. coli* [ST-131]	*aac(6′)Ib*-cr-like, *aac*A4-like, *aad*A1, *bla*_ACC-1_, *bla*_CMY-2_, ***bla***_**VIM−1**_, *cat*A1-like, *str*A, *str*B, *sul*1-like	IncI1 [ST-12], IncHI2 [ST-1], IncF [F18:A6:B1]
T_G-336-2_VIM_	–	*E. coli* [ST-1060]	*aac(6′)Ib*-cr-like, *aac*A4-like, *aad*A1, *bla*_ACC-1_, ***bla***_**VIM−1**_, *cat*A1-like, *str*A, *str*B, *sul*1-like	IncHI2 [ST-1]
T_G-336-2_VIM+CMY_	–	*E. coli* [ST-1060]	*aac(6′)Ib*-cr-like, *aac*A4-like, *aad*A1, *bla*_CMY-2_, ***bla***_**VIM−1**_, *cat*A1-like, *sul*1,	IncI1 [ST-12]
T_G336-2_CMY_	–	*E. coli* [ST-1060]	*bla*_CMY-2_	IncI1 [ST-12]
G-268-2	54	*E. coli* [ST-354]	*aad*A1, *aad*A2, *aph*(3′)-Ia-like, *bla*_CMY-2_, *bla*_TEM-1*B*_, *cml*A1-like, *dfr*A17, *dfr*A8, *mcr*-1, *str*A, *str*B, *sul*2, *sul*3, *tet*(B)	ColRNAI, IncX1, p0111, IncX4, IncQ1, IncB/O/K/Z, IncF [F-:A1:B1]
**RECIPIENT**				
NEB10-beta		*E. coli* [ST-1060]	None	None

* *Previously identified by Fischer et al. (2013a); carbapenemase genes in bold print*.

### The carbapenem resistance of the *E. coli* isolate G-268-2 remains unclear

In case of the second *E. coli* isolate G-268-2 (ST-354) neither ResFinder (Zankari et al., 2012) nor RGI (Jia et al., 2017) indicated the presence of a known carbapenemase gene. Data assembly using two different algorithms (SPAdes and A5-miseq) provided identical results. Besides several other antibiotic resistance genes, this isolate contained the colistin resistance gene *mcr-1* encoded on an IncX4 plasmid (Table 1). Resistance to colistin (MIC = 8mg/L) was confirmed for G-268-2 and its transformant T_G-268-2_mcr-1_ (Table 2). Moreover, the increased MIC for meropenem (4 mg/L) and imipenem (MIC = 8 mg/L) in another G-268-2 transformant indicated a transferrable carbapenem resistance. While the plasmid-encoded colistin resistance was transferred separately (IncX4 plasmid), the reduced carbapenem susceptibility was detected in the transformant containing the AmpC-β-lactamase encoding gene *bla*_CMY-2_ located on an IncB/O/K/Z-plasmid (Table 2). A sequence comparison of the 83,592 kb contig containing the *bla*_CMY-2_ gene of G-268-2 with available plasmid sequences in the GenBank database exhibited 99.98% nucleotide sequence identity (97.3% coverage) to the IncK2 plasmid pDV45 (KR905384) from an *E. coli* isolate from poultry meat (Seiffert et al., 2017). A minor difference was observed in the organization within the shufflon region of *pilV*.

**Table 2:** Antibiotic susceptibilities of wildtype strains and tranformants.

**Farm/region**	**Wildtype isolate/transformants (T)**	**Species - ST**	**PIP**	**PTZ**	**CTX**	**CAZ**	**FEP**	**ATM**	**IMI**	**MEM**	**AMK**	**GEN**	**TBM**	**CIP**	**TGC**	**FOS**	**CST**	**SXT**
1/east	R3 (Fischer et al., 2013a)	*Salmonella* Infantis- ST32	≥128	≥128	≥64	≥64	8	≤1	8	≥16	≤2	2	8	0.5	≤0.5	≤16	≤0.5	≥320
54/east	G-268-2	*E. coli*- ST354	≥128	≥128	≥64	≥64	8	≥64	≥16	8	4	≤1	2	≥4	≤0.5	≤16	8	≥320
	T_G268-2_CMY_	*E. coli*- ST1060	≥128	≥128	≥64	≥64	16	≥64	8	4	4	≤1	≤1	≤0.25	≤0.5	≤16	≤0.5	≤20
	T_G268-2_Mcr-1_	*E. coli*- n.d.	≤4	≤4	≤1	≤1	≤1	≤1	≤0.25	≤0.25	≤2	≤1	≤1	≤0.25	≤0.5	≤16	8	≤20
78/south	G-336-1a	*Salmonella* subspecies I- ST32	64	64	32	≥64	≥64	≤1	≥16	disc.	≤2	≤1	2	0.5	≤0.5	≤16	≤0.5	≤20
	T_G336-1*a*_	*E. coli*- ST1060	≥128	≥128	≥64	≥64	16	≤1	8	8	≤2	≤1	4	0.5	≤0.5	≤16	≤0.5	≤20
78/ south	G-336-2	*E. coli*- ST131	≥128	≥128	≥64	≥64	16	16	≥16	≥16	≤2	≤1	8	1	≤0.5	≤16	≤0.5	≤20
	T_G336-2_VIM_	*E. coli*- ST1060	≥128	≥128	≥64	≥64	16	≤1	4	disc.	≤2	≤1	8	0.5	≤0.5	≤16	≤0.5	≤20
	T_G-336-2_VIM+CMY_	*E. coli*- ST1060	≥128	≥128	≥64	≥64	≥64	≥64	8	≥16	≤2	4	≥16	0.5	≤0.5	≤16	≤0.5	≤20
	T_G336-2_CMY_	*E. coli*- ST1060	≥128	≥128	≥64	≥64	2	≥64	0.5	≤0.25	≤2	≤1	≤1	≤0.25	≤0.5	≤16	≤0.5	≤20
**RECIPIENTS**																		
	NEB10-beta	*E. coli*- ST1060	≤4	≤4	≤1	≤1	≤1	≤1	≤0.25	≤0.25	≤2	≤1	≤1	≤0.25	≤0.5	≤16	≤0.5	≤20
	NEB5-alpha	*E. coli*- n.d.	≤4	≤4	≤1	≤1	≤1	≤1	≤0.25	≤0.25	≤2	≤1	≤1	≤0.25	≤0.5	≤16	≤0.5	≤20

*PIP, piperacillin; PTZ, piperacillin-tazobactam; CTX, cefotaxime; CAZ, ceftazidime; FEP, cefepime; ATM, aztreonam; IPM, imipenem; MEM, meropenem; AMK, amikacin; GEN, gentamicin; TBM, tobramycin; CIP, ciprofloxacin; TGC, tigecycline; FOF, fosfomycin; CST, colistin; SXT, trimethoprim-sulfamethoxazole*.

*disc., run discontinued; n.d., not determined; ST, sequence type*.

The *bla*_CMY-2_ gene itself was located within a mobile genetic element consisting of IS*Ecp1 – bla*_CMY-2_ – *blc* and *sug*E1 as described by Seiffert et al. (2017).

As previously the correlation between carbapenem resistance and the combination of elevated CMY-2-production and porin deficiency has been shown (Goessens et al., 2013; van Boxtel et al., 2017), the whole genome data of the G-268-2 wildtype isolate were additionally checked for mutations in associated regions. Neither the *bla*_CMY-2_ gene nor its promotor region showed any kind of modification. However, the average read coverage of the *bla*_CMY-2_ containing contig was 8.5-fold higher than the coverage of chromosomal contigs. Analysis of the plasmid copy number controlling antisense *inc*RNAI upstream of *repA* showed a nucleotide substitution comparing to the *inc*RNAI of plasmid pDV45 (KR905384) (Seiffert et al., 2017). Analysis by RNA folding prediction software (RNAfold WebServer) indicated that this nucleotide substitution might have an impact on the folding structure of the antisense *inc*RNA (Supplementary Figure 1).

Mapping of the whole genome reads of G-268-2 against the *E. coli* K12 porin genes *omp*F as well as *omp*C exhibited a deletion of 19 nucleotides at nucleotide position 249 in the *ompC* gene, resulting in a frameshift and a premature stop codon at nucleotide position 285. *OmpF* showed 48 silent nucleotide substitutions and 22 nucleotide substitutions leading to amino acid substitutions and exhibited a deletion of 15 nucleotides in comparison to the *ompF* gene of *E. coli* K-12 MG1655 (NC_000913.3). Subsequently performed PCR and Sanger sequencing of the *omp*F- and *omp*C-products supported this observation in both genes. As a control, the mapping of the whole genome reads of the recipient strain NEB10®-beta against both K12 *omp*-genes was performed and both of them were unaltered according to the reference. In contrast to G-268-2, 100% accordance to the *ompF* and *ompC* reference sequences (*E. coli* K12 MG1655) was detected for the transformant T_G-268-2_. Moreover, the additionally performed Blue-Carba assay clearly indicated imipenem-hydrolyzing activity in case of the investigated wild-type isolate G-268-2 as well as its transformant T_G-268-2_CMY-2_ (Supplementary Figure 2).

### Investigation of additional isolates derived from the initially VIM-1-positive tested chicken farm 1 did not possess additional carbapenemase-producing isolates

The retrospective analysis of 125 bacterial isolates derived from three different samplings in chicken farm 1, revealed no additional VIM-1-producing isolates. Therefore, the *S*. Infantis (R3) containing dust sample, which was included in analysis as a positive control remained the only *bla*_VIM-1_ positive sample in the investigated stable.

## Discussion

Although the here described study is based on a retrospective investigation of bacterial isolates sampled in the years 2011 and 2012, the finding of additional carbapenemase-producing Enterobacteriaceae within German chicken farms is worrying. Aware that in the same timeframe also *bla*_VIM-1_ positive *E. coli* and/or *S*. Infantis have been isolated from three German pig fattening farms (Fischer et al., 2017), it becomes obvious that the entry of carbapenemase-producing bacteria into livestock farms got on the way some years ago. Although three out of 45 chicken fattening farms (6.6%), harboring carbapenem-resistant bacteria have been identified in 2011/12 it has to be considered that this study was merely based on pooled feces, boot swabs as well as dust samples. Therefore, the received results provide just a brief overview of the situation within the investigated years and as a previously performed reinvestigation on a VIM-1-positive pig-farm has shown, changes within the resistance situation could occur over the years (Roschanski et al., 2016). However, newer publications indicate that the trend of finding carbapenemase-producing bacteria in livestock as well as food did not stop. Three recently published manuscripts described the finding of additional VIM-1-positive isolates in Germany: *E. coli* derived from the colon contents of slaughter pigs (Irrgang et al., 2017), as well as two *S*. Infantis isolated from minced pork meat and a sick piglet (Borowiak et al., 2017). All of the—so far—in Germany detected *bla*_VIM-1_ encoding livestock associated *Salmonella* isolates belonged to sequence type ST32 and possessed a highly related plasmid (size 300kb, Figure 1). The latter *S*. Infantis, isolated in 2015 and 2016, showed a major homology to the previously detected *S*. Infantis (ST32) isolates R25 and R27 (pig farms) as well as R3 and G-336-1a (chicken farms). Therefore, the here described data as well as previous findings within the pig-production chain suggested a broad circularization in livestock animals (pig- as well as poultry). As in Germany the treatment of livestock with carbapenems is not licensed, a co-selection process over the years seemed to be most likely to explain the consistent re-occurrence of highly similar isolates or plasmids over the years. Moreover an additional report depicted the finding of a *bla*_VIM-1_ positive *E. coli* from a venus clam derived from a Berlin retail market (Roschanski et al., 2017b). However, compared to the *E. coli* isolates derived from livestock in this case neither a strain- nor a plasmid homology has been detected.

Though, not only in Germany an increased finding of carbapenemase producers in livestock or food has been described. *Klebsiella* containing *bla*_NDM_ have been detected in Egypt chicken retail meat and 35% of investigated Egyptian broilers were carrying NDM-, KPC- and or OXA-48- producing *Klebsiella pneumoniae* (Hamza et al., 2016). In 2017, the number of reports was even higher: VIM-positive *Pseudomonas* species were isolated from chicken and their surroundings in China (Zhang et al., 2017). Moreover, in China *bla*_NDM−5_ positive *K. pneumoniae* have been detected in dairy cows, while *bla*_NDM_-producing *E. coli* have been identified in piglets housed in India (Pruthvishree et al., 2017). Furthermore, in the USA *bla*_IMP−27_ containing Enterobacteriaceae were recovered from the environment of a swine farrow-to-finish operation (Mollenkopf et al., 2016). This increase within the last 6 years is alarming and the development of intervention strategies are urgently needed to curtail a further spread of these bacteria. However, beside the bacteria themselves also mobile genetic elements like plasmids or transposons play an important role for the spread of carbapenemase genes. Like here described, farm 78 harbored an *E. coli* as well as a *S*. Infantis (serological typed as *S*. subspecies I with a rough phenotype), carrying the ca. 300 bp IncHI2 plasmid encoding the *bla*_VIM-1_ gene which was previously described for different *S*. Infantis isolates (Fischer et al., 2013a; Borowiak et al., 2017; Falgenhauer et al., 2017). Moreover, our *in-vitro* experiments demonstrated that the Tn*21-like* transposon harboring the *bla*_VIM-1_ gene was able to change its localization from the ca. 300 kb IncHI2 plasmid to a much smaller (ca. 100 kb) IncI1 plasmid. If this event happens in the farm surrounding, it might contribute to the spread of this carbapenemase gene even more efficiently. The exclusive finding of the VIM-1-producing isolates in dust samples of the two farms might be due to the high survival rates of *Salmonella* species in dust or dried manure. In 2015, viable *Salmonella* were detected for up to 291 days in manure dust with 5% moisture (Oni et al., 2015). The fact that the *bla*_VIM-1_-containing isolates did not spread throughout the 2011 and 2012 investigated stables can be explained when the antimicrobial treatment of the two respective flocks is taken into account: While in farm 1 the animals remained untreated during the whole fattening period, in farm 78 an oral treatment with colistin-sulfate was performed for 4 days. As in both cases, no selection pressure was present to favor the spread of the carbapenemase gene-carrying plasmid; this might be a possible explanation for the rare finding of positive tested isolates. However, as the survival rate of *Salmonella* seemed to be pretty high within manure dust (Oni et al., 2015), follow-up investigations of the respective stables would have been desirable.

In addition, the presence of a transferable carbapenem resistance has been shown for the *E. coli* isolate G-268-2 and the assumed presence of a carbapenemase was supported by the Blue-Carba assay (Pires et al., 2013), indicating an imipenem-hydrolyzing activity (Supplementary Figure 2), however, no known carbapenemase gene has been detected within the whole genome data. However, Mammeri et al. reported a higher overall catalytic activity of CMY-2 for imipenem compared to the ones of other pAmpCs (Mammeri et al., 2010). Moreover, several publications depicted the combination of elevated *bla*_CMY-2_ expression caused by an increased plasmid copy number in combination with the lack of the outer membrane proteins OmpC and OmpF as a main reason for detected carbapenem resistance without the presence of a carbapenemase gene in *E. coli* isolates (Chia et al., 2009; Mammeri et al., 2010; Goessens et al., 2013; van Boxtel et al., 2017). In 2012 Kurpiel et al. reported that point mutations in the *inc* antisense RNA gene can be associated with an increased plasmid copy number and subsequently higher expression of *bla*_CMY-2_ (Kurpiel and Hanson, 2012). The observed nucleotide substitution in the *inc* RNA gene of plasmid pG-268-2_CMY-2, however, might be associated with a change in the RNA folding of the Inc antisense RNA and hence led to an increased copy number of this plasmid due to inhibited pseudoknot formation (Supplementary Figure 1). Additional studies will be necessary to check this hypothesis *in-situ*.

Regarding the outer membrane composition, a loss of OmpC was found in isolate G-268-2, while the amount of mutations within the *ompF* gene sequence in the same way suggested a malfunctioned outer membrane protein F.

However, as mentioned previously, the phenotypic carbapenem resistance of G-268-2 (MIC_IMI_ = ≥ 16 mg/L; MIC_MEM_ = 8 mg/L) was transferrable to the *E. coli* recipient strain NEB10®-beta (MIC_IMI_ = 8 mg/L; MIC_MEM_ = 4 mg/L) and the *ompF* as well as *ompC* sequence data of the transformant strain (T_G-268-2_) showed 100% accordance to the reference sequences of *E. coli* K12. Furthermore, the G-268-2 wildtype and its transformant have shown the ability to hydrolyze imipenem in the Blue-Carba assay. Due to the fact that on the one hand site T_G-268-2_ did not possess *ompC* as well as *ompF* mutations and in addition the results of the Blue-Carba assay showed its ability to hydrolyze imipenem, the final explanation for the detected carbapenem resistance of the *E. coli* isolate G-268-2 remains unclear. Further analyses addressing the question if an overexpression of CMY-2 or the occurrence of a new carbapenemase gene might have contributed this observation have to be checked in the future.

Taken together, the knowledge that carbapenem-resistant *Salmonella* as well as *E. coli* isolates can be found within German livestock-farms is alarming and it becomes even worse when these isolates acquired plasmids containing the colistin resistance gene *mcr-1*. Once more, it depicts the importance of comprehensive as well as harmonized monitoring programs in Germany as well as abroad. Beyond this, the implementation of proper intervention strategies to prevent a further dissemination of multidrug-resistant bacteria as well as the spread of their mobile genetic elements within and between animals and humans are urgently needed.

## Author contributions

UR and LK: performed the design of the initial studies; NR, JF, BG, YP: designed the experiments; NR, JF: performed the laboratory work; LF, TC, JF, MP: performed whole genome sequencing including the subsequent data analyses; SG: performed MIC determination; BG, YP: provided scientific support regarding the data evaluation; NR: evaluated the final data and wrote the manuscript. All authors have read and approved the final draft of the article.

### Conflict of interest statement

The authors declare that the research was conducted in the absence of any commercial or financial relationships that could be construed as a potential conflict of interest.

## Supplementary material

Supplementary Figure 1 **(A)** Sequence alignment of the incRNAI of IncB (I), IncK2-pDV45 (II) and pG-268-2 (III). The IncB incRNAI reference sequence derived from Siemering et al. (1993), the IncK2-pDV45 incRNAI reference sequence from Seiffert et al. (2017). **(B)** Predicted RNA folding of the incRNAI of IncB (I), IncK2-pDV45 (II) and pG-268-2 (III) by RNAfold web server.

Supplementary Figure 2 Results of the Blue-Carba assay obtained from *E. coli* Isolate G-268-2. Beside the wildtype strain also its transformants containing the IncB/O/K/Z-plasmid (*bla*_CMY-2_) and the IncX4-plasmid (*mcr-1*) were tested. As positive control (PC) the *bla*_VIM-1_ containing isolate R178 was used. NTC, no template control.

## References

[B1] Abdallah H. M. Reuland E. A. Wintermans B. B. Al Naiemi N. Koek A. Abdelwahab A. M.. (2015). Extended-spectrum beta-lactamases and/or carbapenemases-producing enterobacteriaceae isolated from retail chicken meat in zagazig, Egypt. PLoS ONE 10:e0136052. 10.1371/journal.pone.0136052 26284654 PMC4540287

[B2] Alikhan N. F. Petty N. K. Ben Zakour N. L. Beatson S. A. (2011). BLAST Ring Image Generator (BRIG): simple prokaryote genome comparisons. BMC Genomics 12:402 10.1186/1471-2164-12-402 21824423 PMC3163573

[B3] Bankevich A. Nurk S. Antipov D. Gurevich A. A. Dvorkin M. Kulikov A. S.. (2012). SPAdes: a new genome assembly algorithm and its applications to single-cell sequencing. J. Comput. Biol. 19, 455–477. 10.1089/cmb.2012.0021 22506599 PMC3342519

[B4] Bolger A. M. Lohse M. Usadel B. (2014). Trimmomatic: a flexible trimmer for Illumina sequence data. Bioinformatics 30, 2114–2120. 10.1093/bioinformatics/btu170 24695404 PMC4103590

[B5] Borowiak M. Szabo I. Baumann B. Junker E. Hammerl J. A. Kaesbohrer A.. (2017). VIM-1-producing Salmonella Infantis isolated from swine and minced pork meat in Germany. J. Antimicrob. Chemother. 72, 2131–2133 10.1093/jac/dkx101 28369508

[B6] Carattoli A. (2013). Plasmids and the spread of resistance. Int. J. Med. Microbiol. 303, 298–304. 10.1016/j.ijmm.2013.02.001 23499304

[B7] Carattoli A. Zankari E. Garcia-Fernandez A. Larsen M. V. Lund O. Villa L.. (2014). *In silico* detection and typing of plasmids using plasmidfinder and plasmid multilocus sequence typing. Antimicrobial Agents Chemother. 58, 3895–3903. 10.1128/AAC.02412-14 24777092 PMC4068535

[B8] Chia J. H. Siu L. K. Su L. H. Lin H. S. Kuo A. J. Lee M. H.. (2009). Emergence of carbapenem-resistant *Escherichia coli* in Taiwan: resistance due to combined CMY-2 production and porin deficiency. J. Chemother. 21, 621–626. 10.1179/joc.2009.21.6.621 20071284

[B9] Clermont O. Christenson J. K. Denamur E. Gordon D. M. (2013). The Clermont *Escherichia coli* phylo-typing method revisited: improvement of specificity and detection of new phylo-groups. Environ. Microbiol. Rep. 5, 58–65. 10.1111/1758-2229.12019 23757131

[B10] Coil D. Jospin G. Darling A. E. (2015). A5-miseq: an updated pipeline to assemble microbial genomes from Illumina MiSeq data. Bioinformatics 31, 587–589. 10.1093/bioinformatics/btu661 25338718

[B11] Ewers C. Klotz P. Leidner U. Stamm I. Prenger-Berninghoff E. Gottig S.. (2017). OXA-23 and ISAba1-OXA-66 class D beta-lactamases in *Acinetobacter baumannii* isolates from companion animals. Int. J. Antimicrob. Agents 49, 37–44. 10.1016/j.ijantimicag.2016.09.033 27890443

[B12] Falgenhauer L. Ghosh H. Guerra B. Yao Y. Fritzenwanker M. Fischer J.. (2017). Comparative genome analysis of IncHI2 VIM-1 carbapenemase-encoding plasmids of *Escherichia coli* and *Salmonella enterica* isolated from a livestock farm in Germany. Vet. Microbiol. 200, 114–117. 10.1016/j.vetmic.2015.09.001 26411323

[B13] Fischer J. Rodriguez I. Schmoger S. Friese A. Roesler U. Helmuth R.. (2013a). *Salmonella enterica* subsp. enterica producing VIM-1 carbapenemase isolated from livestock farms. J. Antimicrob. Chemother. 68, 478–480. 10.1093/jac/dks393 23034713

[B14] Fischer J. San Jose M. Roschanski N. Schmoger S. Baumann B. Irrgang A.. (2017). Spread and persistence of VIM-1 Carbapenemase-producing Enterobacteriaceae in three German swine farms in 2011 and 2012. Vet. Microbiol. 200, 118–123. 10.1016/j.vetmic.2016.04.026 27234907

[B15] Fischer J. Schmoger S. Jahn S. Helmuth R. Guerra B. (2013b). NDM-1 carbapenemase-producing *Salmonella enterica* subsp. enterica serovar Corvallis isolated from a wild bird in Germany. J. antimicrob. Chemother. 68, 2954–2956. 10.1093/jac/dkt260 23818284

[B16] Goessens W. H. F. van der Bij A. K. van Boxtel R. Pitout J. D. D van Ulsen P. Melles D. C.. (2013). Antibiotic trapping by plasmid-encoded CMY-2 beta-lactamase combined with reduced outer membrane permeability as a mechanism of carbapenem resistance in *Escherichia coli*. Antimicrob. Agents Chemother. 57, 3941–3949. 10.1128/AAC.02459-12 23733461 PMC3719783

[B17] Gruber A. R. Lorenz R. Bernhart S. H. Neubock R. Hofacker I. L. (2008). The Vienna RNA websuite. Nucleic Acids Res. 36, W70–W74. 10.1093/nar/gkn188 18424795 PMC2447809

[B18] Guerra B. Junker E. Miko A. Helmuth R. Mendoza M. C. (2004). Characterization and localization of drug resistance determinants in multidrug-resistant, integron-carrying *Salmonella enterica* serotype Typhimurium strains. Microb. Drug Resist. 10, 83–91. 10.1089/1076629041310136 15256022

[B19] Hamza E. Dorgham S. M. Hamza D. A. (2016). Carbapenemase-producing Klebsiella pneumoniae in broiler poultry farming in Egypt. J. Glob. Antimicrob. Resist. 7, 8–10. 10.1016/j.jgar.2016.06.004 27530998

[B20] He T. Wang Y. Sun L. Pang M. Zhang L. Wang R. (2017). Occurrence and characterization of blaNDM-5-positive Klebsiella pneumoniae isolates from dairy cows in Jiangsu, China. J. Antimicrob. Chemother. 72, 90–94. 10.1093/jac/dkw357 27621177

[B21] Hering J. Fromke C. von Munchhausen C. Hartmann M. Schneider B. Friese A.. (2016). Cefotaxime-resistant *Escherichia coli* in broiler farms-A cross-sectional investigation in Germany. Prev. Vet. Med. 125, 154–157. 10.1016/j.prevetmed.2016.01.003 26783199

[B22] Irrgang A. Fischer J. Grobbel M. Schmoger S. Skladnikiewicz-Ziemer T. Thomas K.. (2017). Recurrent detection of VIM-1-producing Escherichia coli clone in German pig production. J. Antimicrob. Chemother. 72, 944–946. 10.1093/jac/dkw479 28007897 PMC5400094

[B23] Jia B. Raphenya A. R. Alcock B. Waglechner N. Guo P. Tsang K. K.. (2017). CARD 2017: expansion and model-centric curation of the comprehensive antibiotic resistance database. Nucleic Acids Res. 45, D566–D573. 10.1093/nar/gkw1004 27789705 PMC5210516

[B24] Kaas R. S. Leekitcharoenphon P. Aarestrup F. M. Lund O. (2014). Solving the problem of comparing whole bacterial genomes across different sequencing platforms. PLoS ONE 9:e104984. 10.1371/journal.pone.0104984 25110940 PMC4128722

[B25] Kurpiel P. M. Hanson N. D. (2012). Point mutations in the inc antisense RNA gene are associated with increased plasmid copy number, expression of blaCMY-2 and resistance to piperacillin/tazobactam in *Escherichia coli*. J. Antimicrob. Chemother. 67, 339–345. 10.1093/jac/dkr479 22117029

[B26] Larsen M. V. Cosentino S. Rasmussen S. Friis C. Hasman H. Marvig R. L.. (2012). Multilocus sequence typing of total-genome-sequenced bacteria. J. Clin. Microbiol. 50, 1355–1361. 10.1128/JCM.06094-11 22238442 PMC3318499

[B27] Lartigue M. F. Poirel L. Poyart C. Reglier-Poupet H. Nordmann P. (2007). Ertapenem resistance of *Escherichia coli*. Emerg. Infect. Dis. 13, 315–317. 10.3201/eid1302.060747 17479901 PMC2725854

[B28] Laube H. Friese A. von Salviati C. Guerra B. Kasbohrer A. Kreienbrock L. (2013). Longitudinal monitoring of Esbl/Ampc-Producing *Escherichia coli* in German broiler chicken fattening farms. Appl. Environ. Microbiol. 79, 4815–4820 10.1128/AEM.00856-13 23747697 PMC3754693

[B29] Mammeri H. Guillon H. Eb F. Nordmann P. (2010). Phenotypic and biochemical comparison of the carbapenem-hydrolyzing activities of five plasmid-borne AmpC beta-lactamases. Antimicrob. Agents Chemother. 54, 4556–4560. 10.1128/AAC.01762-09 20733047 PMC2976168

[B30] Mollenkopf D. F. Stull J. W. Mathys D. A. Bowman A. S. Feicht S. M. Grooters S. V.. (2016). Carbapenemase-producing Enterobacteriaceae recovered from the environment of a swine farrow-to-finish operation in the United States. Antimicrob. Agents Chemother. 61:aac.02348-16. 10.1128/AAC.01298-16 27919894 PMC5278694

[B31] Morrison B. J. Rubin J. E. (2015). Carbapenemase producing bacteria in the food supply escaping detection. PLoS ONE 10:e0126717. 10.1371/journal.pone.0126717 25966303 PMC4429064

[B32] Nordmann P. Dortet L. Poirel L. (2012). Carbapenem resistance in Enterobacteriaceae: here is the storm! Trends Mol. Med. 18, 263–272. 10.1016/j.molmed.2012.03.003 22480775

[B33] Olasz F. Nagy T. Szabo M. Kiss J. Szmolka A. Barta E. (2015). Genome sequences of three *Salmonella enterica* subsp. enterica Serovar Infantis strains from healthy broiler chicks in Hungary and in the United Kingdom. Genome announc. 3:e01468–14. 10.1128/genomeA.01468-14 25676749 PMC4333649

[B34] Oni R. A. Sharma M. Buchanan R. L. (2015). Survival of *Salmonella enterica* in dried Turkey manure and persistence on spinach leaves. J. Food Prot. 78, 1791–1799. 10.4315/0362-028X.JFP-15-047 26408127

[B35] Grimont P. A. D. Weill F.-X. (2007). Antigenic Formulae of the Salmonella Serovars WHO Collaborating Centre for Reference and Research on Salmonella, 9th Edn Paris: Institut Pasteur.

[B36] Pires J. Novais A. Peixe L. (2013). Blue-carba, an easy biochemical test for detection of diverse carbapenemase producers directly from bacterial cultures. J. Clin. Microbiol. 51, 4281–4283. 10.1128/JCM.01634-13 24108615 PMC3838089

[B37] Pruthvishree B. S. Vinodh Kumar O. R. Sinha D. K. Malik Y. P. S. Dubal Z. B. Desingu P. A.. (2017). Spatial molecular epidemiology of carbapenem-resistant and New Delhi metallo beta-lactamase (blaNDM)-producing *Escherichia coli* in the piglets of organized farms in India. J. Appl. Microbiol. 122, 1537–1546. 10.1111/jam.13455 28345184

[B38] Ribot E. M. Fair M. A. Gautom R. Cameron D. N. Hunter S. B. Swaminathan B.. (2006). Standardization of pulsed-field gel electrophoresis protocols for the subtyping of *Escherichia coli* O157:H7, Salmonella, and Shigella for PulseNet. Foodborne Pathog. Dis. 3, 59–67. 10.1089/fpd.2006.3.59 16602980

[B39] Roschanski N. Friese A. von Salviati-Claudius C. Hering J. Kaesbohrer A. Kreienbrock L.. (2017a). Prevalence of carbapenemase producing Enterobacteriaceae isolated from German pig-fattening farms during the years 2011-2013. Vet. Microbiol. 200, 124–129. 10.1016/j.vetmic.2015.11.030 26654218

[B40] Roschanski N. Friese A. Thieck M. Roesler U. (2016). Follow-up investigation of the first VIM-1 positive pig farm in Germany - How is the situation 4 years after the first detection? Clin. Microbiol. Infect. 22, 951–953. 10.1016/j.cmi.2016.08.018 27596533

[B41] Roschanski N. Guenther S. Vu T. T. T. Fischer J. Semmler T. Roesler U.. (2017b). VIM-1 carbapenemase-producing *Escherichia coli* isolated from retail seafood, Germany 2016. Euro Surveill. 22, 1 7:32. 10.2807/1560-7917.ES.2017.22.43.17-00032 29090680 PMC5718389

[B42] Rubin J. E. Ekanayake S. Fernando C. (2014). Carbapenemase-producing organism in food, 2014. Emerg. Infect. Dis. 20, 1264–1265. 10.3201/eid2007.140534 24960459 PMC4073846

[B43] Seiffert S. N. Carattoli A. Schwendener S. Collaud A. Endimiani A. Perreten V. (2017). Plasmids carrying blaCMY−2/4 in *Escherichia coli* from poultry, poultry meat, and humans belong to a novel IncK subgroup designated IncK2. Front. Microbiol. 8:407. 10.3389/fmicb.2017.00407 28360894 PMC5350095

[B44] Siemering K. R. Praszkier J. Pittard A. J. (1993). Interaction between the antisense and target RNAs involved in the regulation of IncB plasmid replication. J. Bacteriol. 175, 2895–2906. 7684039 10.1128/jb.175.10.2895-2906.1993 PMC204607

[B45] Stolle I. Prenger-Berninghoff E. Stamm I. Scheufen S. Hassdenteufel E. Guenther S.. (2013). Emergence of OXA-48 carbapenemase-producing *Escherichia coli* and *Klebsiella pneumoniae* in dogs. J. Antimicrob. Chemother. 68, 2802–2808. 10.1093/jac/dkt259 23833179

[B46] van Boxtel R. Wattel A. A. Arenas J. Goessens W. H. Tommassen J. (2017). Acquisition of carbapenem resistance by Plasmid-Encoded-AmpC-Expressing *Escherichia coli*. Antimicrob. Agents chemother. 61:e01413–16. 10.1128/AAC.01413-16 27799202 PMC5192137

[B47] Walsh T. R. Weeks J. Livermore D. M. Toleman M. A. (2011). Dissemination of NDM-1 positive bacteria in the New Delhi environment and its implications for human health: an environmental point prevalence study. Lancet Infect. Dis. 11, 355–362. 10.1016/S1473-3099(11)70059-7 21478057

[B48] Wang Y. Zhang R. Li J. Wu Z. Yin W. Schwarz S.. (2017). Comprehensive resistome analysis reveals the prevalence of NDM and MCR-1 in Chinese poultry production. Nat. microbiol. 2:16260. 10.1038/nmicrobiol.2016.260 28165472

[B49] Wilson A. P. R. (2017). Sparing carbapenem usage. J. Antimicrob. Chemother. 72, 2410–2417 10.1093/jac/dkx181 28637307

[B50] Zankari E. Hasman H. Cosentino S. Vestergaard M. Rasmussen S. Lund O.. (2012). Identification of acquired antimicrobial resistance genes. J. Antimicrob. Chemother. 67, 2640–2644. 10.1093/jac/dks261 22782487 PMC3468078

[B51] van der Zee A. Roorda L. Bosman G. Fluit A. C. Hermans M. Smits P. H. (2014). Multi-centre evaluation of real-time multiplex PCR for detection of carbapenemase genes OXA-48, VIM, IMP, NDM and KPC. BMC Infect. Dis. 14:27 10.1186/1471-2334-14-27 24422880 PMC3897903

[B52] Zhang R. Liu Z. Li J. Lei L. Yin W. Li M.. (2017). Presence of VIM-Positive pseudomonas species in chickens and their surrounding environment. Antimicrob. Agents Chemother. 61:e00167–17. 10.1128/AAC.00167-17 28438943 PMC5487629

[B53] Zurfluh K. Hachler H. Nuesch-Inderbinen M. Stephan R. (2013). Characteristics of extended-spectrum beta-lactamase- and carbapenemase-producing Enterobacteriaceae isolates from rivers and lakes in Switzerland. Appl. Environ. Microbiol. 79, 3021–3026. 10.1128/AEM.00054-13 23455339 PMC3623138

