# Supplementary figures and images for: Retrospective Analysis of Bacterial Cultures Sampled in German Chicken-Fattening Farms During the Years 2011–2012 Revealed Additional VIM-1 Carbapenemase-Producing Escherichia coli and a Serologically Rough Salmonella enterica Serovar Infantis
Source: Front Microbiol. 2018 Mar 27;9:538. doi: 10.3389/fmicb.2018.00538 (PMC5880886; doi:10.3389/fmicb.2018.00538)

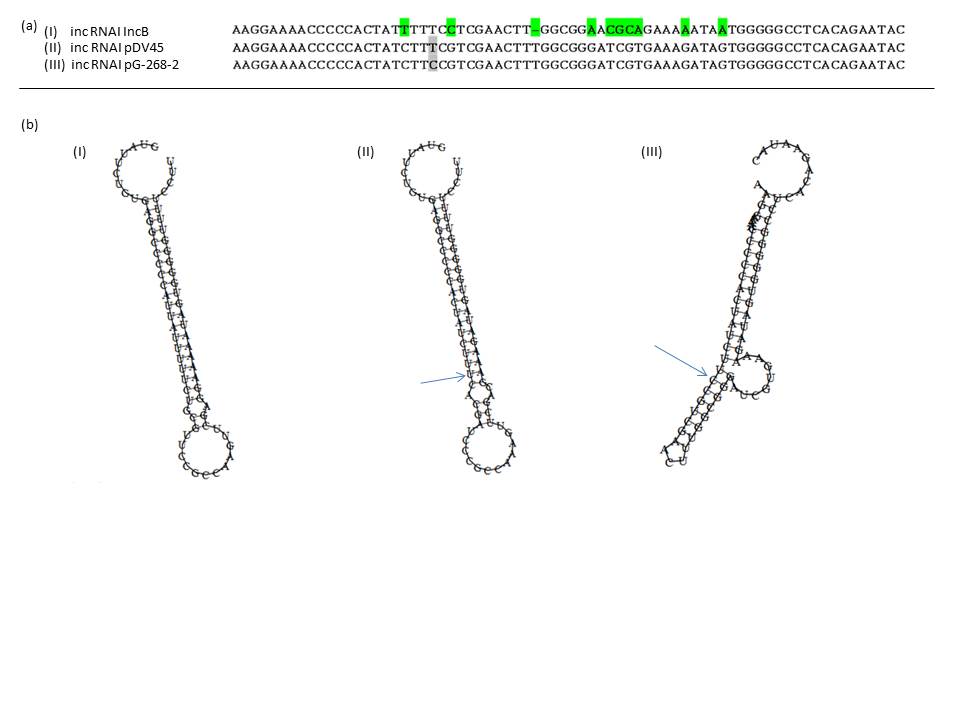

Supplement: Supplementary Figure 1 — (A) Sequence alignment of the incRNAI of IncB (I), IncK2-pDV45 (II) and pG-268-2 (III). The IncB incRNAI reference sequence derived from Siemering et al. (1993), the IncK2-pDV45 incRNAI reference sequence from Seiffert et al. (2017). (B) Predicted RNA folding of the incRNAI of IncB (I), IncK2-pDV45 (II) and pG-268-2 (III) by RNAfold web server. [file Image1.JPEG]

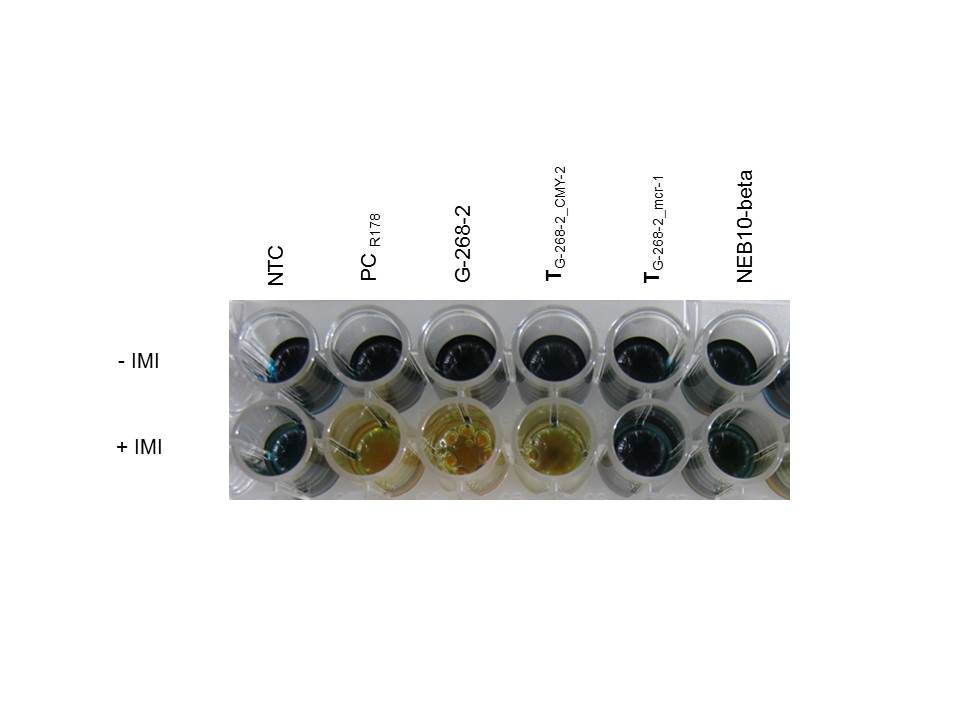

Supplement: Supplementary Figure 2 — Results of the Blue-Carba assay obtained from E. coli Isolate G-268-2. Beside the wildtype strain also its transformants containing the IncB/O/K/Z-plasmid (blaCMY-2) and the IncX4-plasmid (mcr-1) were tested. As positive control (PC) the blaVIM-1 containing isolate R178 was used. NTC, no template control. [file Image2.JPEG]
